# Effects of Kimchi on human health: A protocol of systematic review of controlled clinical trials

**DOI:** 10.1097/MD.0000000000010163

**Published:** 2018-03-30

**Authors:** Myung-Sunny Kim, Hye Jeong Yang, Soon-Hee Kim, Hye Won Lee, Myeong Soo Lee

**Affiliations:** a Division of Nutrition and Metabolism, Korea Food Research Institute; b Department of Food Biotechnology, Korea University of Science and Technology, Jeollabuk-do; c KM Convergence Research Division; d Clinical Research Division, Korea Institute of Oriental Medicine; e Department of Korean Medicine Life Science, Korea University of Science and Technology, Daejeon, Republic of Korea.

**Keywords:** ethnic food, fermented vegetable, health care, Kimchi

## Abstract

**Background:**

Kimchi, a traditional, fermented Korean food that is consumed daily, has been recognized as a health food due to its beneficial effects on human health. The aim of this overview is to critically evaluate all clinical trials of the use of Kimchi in the treatment of any condition or symptom.

**Methods and analysis:**

Eight databases will be searched from inception until March 2018. We will include all prospective trials, including randomized controlled trials (RCTs), non-RCTs, and uncontrolled trials. The methodological quality of the trials will be assessed using Cochrane risk of bias (ROB) assessment tool and ROB in nonrandomized studies-I for RCTs and non-RCTs, respectively.

**Ethics and dissemination:**

Ethical approval will not be required, given that this protocol is for a systematic review. The full systematic review will be published in a peer-reviewed journal. The review will also be disseminated electronically and in print. Updates of the review will be conducted to inform and guide health care practice and policy.

**Trial registration number:**

PROSPERO 2018 CRD42018087375

## 1 Introduction

Kimchi, a traditional, fermented Korean food that is consumed daily, has been recognized as a health food due to its beneficial effects on human health. As it is fermented with various vegetable ingredients and seasonings, it is abundant in dietary fiber, vitamins, lactic acid bacteria, minerals, and other active compounds that are present either in Kimchi ingredients such as cabbage, garlic, ginger, and red pepper powder^[1,2]^ or in fermented products in general. Scientific studies have shown that the biological compounds of Kimchi stimulate immune function and reduce pro-oxidants, free radicals, certain cancers, cardiovascular disease (CVD), metabolic syndrome risks, and aging, as reviewed.^[3–8]^

Several studies have evaluated the potential benefits to human health, and accumulating data on human Kimchi consumption has shown that Kimchi consumption lowers lipids and the atherogenic index in plasma^[9–11]^; it also changes the composition as well as count of intestinal microflora^[12,13]^ and increases iron levels.^[14]^

Since a systematic review of these topics is not available, we access evidence of the beneficial effects of Kimchi consumption on human health. The aim of this overview is to critically evaluate all clinical trials of the use of Kimchi in the treatment of any condition or symptom.

## 2 Methods

### 2.1 Study registration

This protocol of review has been registered on PROSPERO 2018 CRD42018087375 (http://www.crd.york.ac.uk/PROSPERO/display_record.php?ID=CRD42018087375).

### 2.2 Criteria for considering studies for this review

#### 2.2.1 Types of studies

All prospective trials will be included. We will include randomized controlled trials (RCTs) and non-RCTs. We will exclude uncontrolled trials, case series, case studies, and retrospective clinical trials.

#### 2.2.2 Type of participants

We will include trials with healthy persons and persons with any health condition. We will also include trials with participants regardless of age or sex, including children, adults, and the elderly.

#### 2.2.3 Type of interventions

We will include all types of Kimchi, traditional fermented Korean food that is consumed daily, regardless of vegetable ingredients, origin of region, production date, and presence or absence of water.

#### 2.2.4 Type of outcome measures

The primary outcome will be related to the target conditions. We will use the timing and effect measures as the value of the end of treatment. Secondary outcomes will be quality of life and adverse events.

### 2.3 Search methods for the identification of studies

#### 2.3.1 Data searches

The following databases will be searched for content dated up to March 2018: MEDLINE, Embase, and the Cochrane Library, as well as Korean databases (Oriental Medicine Advanced Searching Integrated System, the National Digital Science Library, DBpia, the Research Information Service System, and the Korean Studies Information Service System). Relevant journals include Preventive Nutrition and Food Science, and Food Industry and Nutrition Conference will be hand-searched as gray literature for proceedings from the Korean Society of Food Science and Nutrition. The references in all identified articles will also be searched.

#### 2.3.2 Search strategies

The search strategy will use the key words [“Kimchi” OR “Kim Chi” OR “fermented vegetable”] AND [“clinical trial”] and will be principally based on the individual database-specific structure.

### 2.4 Selection of studies

Two authors (MSK and HJY) will select articles to include by checking article titles and abstracts. These authors will review hard copies of relevant publications to determine their inclusion. Any disagreement will be resolved through discussion and, if necessary, will use a 3rd reviewer (MSL).

### 2.5 Data extraction and management

Three authors will participate in the data extraction (MSK, SHK, and HWL). Information regarding study design, population, symptom types, and interventions and outcomes will be extracted from the studies. Disagreements will be resolved by discussion between the authors.

### 2.6 Risk of bias assessment

The quality of each study will be assessed on the basis of the study design; to this end, risk of bias (ROB) assessment is used for RCTs.^[15]^ Domains including sequence generation, allocation concealment, blinding of participants, blinding of outcome assessors, incomplete outcome data, selective reporting, and other biases will be assessed, and ROB for each domain will be decided as being low, high, or unclear based on the Cochrane collaboration guideline.

The ROB of non-RCTs will be assessed using the ROB in nonrandomized studies-I tools.^[16]^ This tool evaluates bias from confounding, selection of participation, classification of intervention, deviation from intended intervention, missing data, measurement of outcome, and selection of the reported results. Each domain will be rated as having low, severe, critical, or unclear ROB.

### 2.7 Data synthesis

We will conduct a meta-analysis if enough studies for each symptom are identified: risk ratio (RR) for dichotomous outcomes and mean difference (MD) for continuous outcomes will be assumed to be used as summary estimates. Considering the expected clinical heterogeneity of types of Kimchi, a random effect model will be adopted for the meta-analysis. We will use the *I*^2^ statistic for quantifying inconsistencies across the included studies.^[15]^ A result 50% cut off point would represent substantial heterogeneity. If heterogeneity is observed, subgroup analyses will be conducted according to type of Kimchi, type of control intervention (eg, Western medicine, no treatment, or usual care). We will use GRADEpro GDT software (https://gradepro.org/) to create a summary of findings table.

If more than 10 studies are available, we will conduct funnel plot for publication bias and small study effects using Egger method.^[17]^ Funnel plot asymmetry is certainly not same as publication bias. We will attempt to distinguish the possible reasons for the asymmetry, therefore, included poor methodological quality and true heterogeneity of studies.

If there are not enough RCTs for meta-analysis, we will conduct a narrative review based on the research designs. We will summarize the study characteristics and effectiveness of Kimchi based on the individual results of the included studies.

### 2.8 Ethics and dissemination

Ethical approval is not required, given that this protocol is for a systematic review. The findings of this review will be disseminated widely through peer-reviewed publications and conference presentations.

## 3 Discussion

No systematic review of these clinical studies has been conducted yet. The completed review will provide a summary of the current state of evidence and will be useful to researchers, food and nutrition policymakers, and food companies, as well as to patients and consumers.

## Author contributions

**Conceptualization:** M.-S. Kim, M.S. Lee.

**Data curation:** M.-S. Kim.

**Formal analysis:** M.-S. Kim, H.J.Y. Yang.

**Funding acquisition:** M.-S. Kim, M.S. Lee.

**Methodology:** M.S. Lee.

**Supervision:** M.S. Lee.

**Validation:** S.-H. Kim, H.W. Lee.

**Writing – original draft:** M.-S. Kim, M.S. Lee.

**Writing – review & editing:** H.J.Y. Yang, S.-H. Kim, H.W. Lee.
